# Mesoporous Zeolitic Materials (MZMs) Derived From Zeolite Y Using a Microwave Method for Catalysis

**DOI:** 10.3389/fchem.2020.00482

**Published:** 2020-06-30

**Authors:** Samer Abdulridha, Yilai Jiao, Shaojun Xu, Rongxin Zhang, Arthur A. Garforth, Xiaolei Fan

**Affiliations:** ^1^Department of Chemical Engineering and Analytical Science, School of Engineering, The University of Manchester, Manchester, United Kingdom; ^2^Shenyang National Laboratory for Materials Science, Institute of Metal Research, Chinese Academy of Sciences, Shenyang, China

**Keywords:** mesoporous materials, microwave irradiation, post-synthetic treatment, zeolite, MCM-41, catalysis

## Abstract

Mesostructured zeolitic materials (MZMs) with relatively high acidity in comparison with the mesoporous siliceous MCM-41 were prepared via an efficient, mild, and simple post-synthetic treatment of Y zeolite facilitated by microwave irradiation, i.e., microwave-assisted chelation (MWAC). The disordered mesoporous aluminosilicates materials (DMASs) of MZM were created from Y zeolite in the absence of using mesoscale templates. The prepared DMASs showed the good mesoporous features with the mesopore area and volume of ~260 m^2^ g^−1^ and ~0.37 cm^3^ g^−1^, respectively, and with the mesopore sizes distributed in a range of 2–10 nm. MZMs possess a total acidity of about 0.6 mmol g^−1^ and exhibited comparatively superior catalytic activity to the parent Y zeolite and MCM-41 in the vapor phase catalytic dealkylation of 1,3,5-triisopropylbenzene (TiPBz) and liquid phase catalytic aldol condensation of benzaldehyde with 1-heptanal. Although the yield loss was inevitable for preparing MZMs using the MWAC method, the preliminary economic analysis of the preparation cost of MZMs showed the promise. Additionally, a comprehensive comparison of the state-of-the-art mesoporous materials concerning their sustainable aspects was made, showing that MZMs are promising mesoporous materials for further development and functionalization for catalysis.

## Introduction

Mesoporous materials are an important class of porous materials with a wide range of proposed applications in catalysis (particularly as the additives for petrochemical conversions, especially large hydrocarbons), environmental remediation (e.g., adsorbents), biomedical application (e.g., drug delivery), energy storage, and functional devices (e.g., sensors) (Davis, 2002; Sen et al., 2004; Vallet-Regi et al., 2007; Chal et al., 2011; Wang et al., 2016; Fan and Jiao, 2020a). Generally, considering the silica-based mesoporous materials, they can be divided into two classes: ordered mesoporous silica and aluminosilicate materials, such as MCM-41, MCM-48, SBA-15, and MAS-5 (Kresge et al., 1992; Zhao et al., 1998; Zhang et al., 2001; Chaudhary and Sharma, 2016) and disordered mesoporous materials, such as KIT-1 (Ryoo et al., 1996). Ordered mesoporous MCM-41 silica/aluminosilicates are important functional materials due to their high specific surface area (typically at ~1,000 m^2^ g^−1^), hexagonal arrangement of ordered unidirectional mesopores, and narrow pore size distribution (normally 1.5–10 nm) (Beck et al., 1992; Prasomsri et al., 2015). SBA-15, another class of mesoporous silica/aluminosilicates, has also received much attention due to its uniform hexagonal pores, with large pore diameters of 5 to 30 nm, and the relatively thick pore wall of 3.1–6.4 nm (Zhao et al., 1998). However, although the well-structured mesoporosity is valuable to improve the accessibility and molecular diffusion within the framework, the amorphous nature of mesoporous silica frameworks makes them, generally, hydrothermally less stable compared to the crystalline microporous zeolites. Additionally, they are commonly siliceous, being less effective than aluminosilicate zeolites for the solid acid catalyzed reactions (Perego and Millini, 2013; Prasomsri et al., 2015). To improve the catalytic property of mesoporous silica, incorporation of aluminum in their frameworks was explored (Zhai et al., 2004; Locus et al., 2016; La-Salvia et al., 2017). However, this is challenging and results in the preferable disposition of Al species on mesoporous pore walls rather than insertion to the framework, thus being less effective for catalysis since the Al-O-Si framework structure is responsible for Brønsted acidity (Dědeček et al., 2001; Perego and Millini, 2013). Dědeček et al. (2001) studied the effect of Si/Al composition on the aluminum distribution in MCM-41 aluminosilicates, that is, Al-MCM-41. It was found that, at high Si/Al ratios of ≥ 20, only 20% of Al atoms are incorporated into the framework. Regarding Al-MCM-41 with Si/Al < 20, the inclusion of Al atoms in the framework increased notably. For example, in Al-MCM-41 with Si/Al = 11, about 45% of Al atoms was identified as the framework Al species, but octahedrally coordinated. Therefore, the insertion of Al in MCM-41 (Si/Al ≤ 20) did not result in an increase in Brønsted acidity (Dědeček et al., 2001). Commonly, alkaline media is beneficial to facilitate the incorporation of non-framework Al atoms into the mesoporous frameworks (Liu and Pinnavaia, 2002). Locus et al. (2016) carried out the investigation to improve the acidity of Al-MCM-41 via the aluminum activation using alkaline treatment with aqueous NaOH and NH_4_OH solutions. Based on the findings of the Al magic angle spinning nuclear magnetic resonance (MAS NMR) spectroscopy, the method showed the capability of converting octahedral Al to tetrahedral Al, doubling the proportion of the tetrahedral Al species in the activated sample (at ~60%) in comparison with that of the parent Al-MCM-41 aluminosilicate (~30%). The activated Al-MCM-41 demonstrated improved catalytic activity compared with the parent Al-MCM-41 in alkylation of toluene with benzyl alcohol, i.e., a 4-fold increase in the conversion, 32 vs. 8% (Locus et al., 2016). However, the effective acidity of the activated Al-MCM-41 is still relatively low. Efforts have also been made to incorporate Al species in the framework of SBA-15 to improve its acidity (Han et al., 2001; Li et al., 2004). However, it is very difficult to prepare SBA-15 containing framework Al atoms due to the strong acidic conditions employed for synthesizing SBA-15 (pH values at ~1) (Han et al., 2001; Liu and Pinnavaia, 2002; Dos Santos et al., 2013).

Disordered mesoporous silicates with three-dimensional frameworks, such as KIT-1 were also developed and generally showed slightly better hydrothermal (i.e., under boiling water and steaming conditions) stability (Ryoo et al., 1996) than the ordered hexagonal analogs of MCM-41 and MCM-48 (Kim and Ryoo, 1996). However, the disordered mesoporous materials are still of limited use owing to the absence of active sites. Accordingly, based on the disordered mesoporous silica, relevant composite materials were developed with the improved acidity. For instance, the hybrid Y zeolite-assembled MSU-type materials (or Al-MSU-S) were developed, which was prepared by assembling zeolite Y seeds in the hexagonal mesoporous MSU materials at pH values of about 9 in the presence of a surfactant (i.e., cetyltrimethylammonium bromide, CTAB) (Liu et al., 2000). Framework Al species can be varied in a range of 0.01–38 mol% in the Al-MSU-S materials. Although the strategy of using zeolite seeds as precursors for the assembly of aluminosilicates mesostructures was demonstrated generically, the use of mesoscale templates, such as cationic surfactants and triblock copolymers is inevitable (Zhao et al., 2001; Perego and Millini, 2013), hence it is not ideal for applications on a large scale.

The direct synthesis of mesoporous aluminosilicates with ordered or disordered structures was also developed using various templating methods. For example, ordered hexagonal mesoporous aluminosilicates, such as MAS-5 (~2.7 nm pore size) (Zhang et al., 2001) and MAS-7 (~7.6 nm pore size) (Han et al., 2001) were synthesized by assembling Beta-type aluminosilicate precursors using templates, such as CTAB and Pluronic P123. Many DMASs were also prepared using direct synthesis methods employing the aluminosilicate precursors and organic structure-directing agents (e.g., CTAB, P123, and F127) (Lee et al., 2008; Pega et al., 2009; Skoda et al., 2016). Although the ordered/disordered mesoporous aluminosilicates prepared by the strategies discussed above exhibited acidity and improved hydrothermal stability to various extents, the synthesis procedures are generally time-consuming and are potentially not economical, sustainable, or environmentally friendly, specifically concerning the use of templates. The relevant costs associated with the mesoscale templates make the practical large-scale preparation of these mesoporous aluminosilicates not very economical. More importantly, the template removal processes via calcination also indicate significant environmental and economic issues, i.e., toxic flue gas emissions and their treatments (Moller and Bein, 2013; Serrano et al., 2013). Therefore, the development of purely mesostructured aluminosilicate materials for catalysis is still of interest, taking into account not only the specific mesoporous and acidic properties, but also the avoidance of the aforementioned disadvantages experienced in the current practice of making the state-of-the-art mesoporous aluminosilicates.

Herein, we report the preparation of a class of mesoporous zeolitic materials (MZMs) using the developed post-synthetic treatment, i.e., microwave-assisted chelation (MWAC) (Fan and Jiao, 2020b; Zhang et al., 2020), of a commercial Y zeolite. The resulting MZMs were compared with the mesoporous siliceous MCM-41 regarding their mesoporous and acidic features. The catalytic activity of MZM was evaluated using the vapor phase catalytic dealkylation of 1,3,5-triisopropylbenzene (TiPBz) and the liquid-phase catalytic aldol condensation of benzaldehyde with 1-heptanal, using the microporous Y zeolite and mesoporous siliceous MCM-41 as the control catalysts. Additionally, aspects regarding the relevant cost and energy consumption associated with the method of preparing MZMs were also studied preliminarily based on the available data on the laboratory scale.

## Experimental Section

### Chemicals and Materials

The parent zeolite used for the preparation of MZMs in this work was commercial zeolite Y (CBV 300 by Zeolyst International, NH_4_-form, Si/Al = 2.6). Chemicals used by the post-synthetic MWAC treatment include ethylenediaminetetraacetic acid (EDTA, 99%, Sigma-Aldrich), sodium hydroxide (NaOH, 99%, Sigma-Aldrich), and ammonium nitrate (NH_4_NO_3_, ACS reagent ≥98%, Sigma-Aldrich). Mesoporous bulk MCM-41 silica (hexagonal) was purchased from Sigma-Aldrich.

Chemicals used for catalytic aldol condensation and gas chromatography (GC) calibration are benzaldehyde (ReagentPlus®, ≥99%, Sigma-Aldrich), 1-heptanal (97%, Alfa Saesar), dodecane (ReagentPlus®, ≥99%, Sigma-Aldrich), α-amylcinnamaldehyde (jasmin aldehyde, 97%, Sigma-Aldrich), and ethanol (99.7–100% absolute, VWR International).

Chemicals used for catalytic dealkylation of 1,3,5-triisopropylbenzene (TiPBz) and GC calibration are benzene (C_6_H_6_, ≥99.8% Sigma-Aldrich), toluene (C_6_H_5_CH_3_, ≥99.5%, Sigma-Aldrich), *para*-xylene [C_6_H_4_(CH_3_)_2_, ≥99.5% GC, Sigma-Aldrich], *ortho*-xylene [C_6_H_4_(CH_3_)_2_, ≥99.5% GC, Sigma-Aldrich], *meta*-xylene [C_6_H_4_(CH_3_)_2_, ≥99.5% GC, Sigma-Aldrich], cumene (C_9_H_12_, 99%, Alfa Aesar), 1,2,3-trimethylbenzene [C_6_H_3_(CH_3_)_3_, ≥99.5%, neat, GC Sigma-Aldrich], 1,2,4-trimethylbenzene [C_6_H_3_(CH_3_)_3_, 98%, Sigma-Aldrich], 1,3-diisopropylbenzene (C_12_H_18_, 96%, Sigma-Aldrich), 1,4-diisopropylbenzene (C_12_H_18_, 99%, Alfa Aesar), and 1,3,5-triisopropylbenzene (C_15_H_24_, 95%, Alfa Aesar). All chemicals were used as received without further purification.

### Preparation of MZMs via Post-synthetic Treatments of Y Zeolite Under Microwave Irradiation

The development of MWAC method for the post-synthetic treatment of Y zeolite has been described elsewhere (Fan and Jiao, 2020b; Zhang et al., 2020). The experimental details of the MWAC condition used in this work were: 25 mL 0.2 M EDTA solution, zeolite-to-solution ratio = 0.066 g mL^−1^, treatment time = 1 or 30 min, and temperature = 50 and 100°C. MWAC treatment was performed using a CEM Discover SP microwave system at 150 W.

The conventional hydrothermal treatment of zeolite Y using 0.2 M EDTA solution was performed in a 250 mL round-bottom flask (solution volume = 80 mL, zeolite-to-solution ratio = 0.066 g mL^−1^) under reflux for 6 h. The workup procedure of the resulting materials has been detailed elsewhere (Zhang et al., 2020). The samples were named as MZMs and denoted as MZM–*x–y–z*, where *x* refers to the post-synthetic treatment methods, which are MW for the MWAC treatment and HT for the hydrothermal treatment; *y* refers to the treatment time (m for minute and h for hour); and *z* refers to the treatment temperature in degree Celsius (°C), respectively.

### Characterization of Materials

Powder X-ray diffraction (PXRD) patterns of the materials were obtained using a Philips X'Pert X-ray diffractometer with the conditions of Cu*K*α_1_ radiation: λ = 1.5406 Å, 40 kV, 40 mA, 5° < 2θ < 65°, 0.0167° step size. Nitrogen (N_2_) physisorption analysis of the materials was carried out at −196°C using a Micromeritics 3Flex surface characterization analyzer. Prior to N_2_ sorption measurements, all samples (as-prepared, and calcined at 450°C for 5 h) with a weight of ~100 mg were degassed at 350°C under vacuum overnight. Specific surface areas of the catalysts were determined using the Brunauer-Emmett-Teller (BET) method. Pore size analysis was performed using the Barrett-Joyner-Halenda (BJH) method on the adsorption branch of isotherms. X-ray fluorescence (XRF) was performed using PANalytical MiniPal4 (PANalytical EDXRD) spectrometer operated at 30 kV and 1 mA. Scanning electron microscopy (SEM) and energy-dispersive x-ray diffraction (EDX) were undertaken by a FEI Quanta 250 FEG-SEM using a work distance of 8–10 mm and an accelerating voltage of 15 kV. All samples were dispersed in acetone and dropped onto SEM studs, followed by gold deposition using an Emitech K550X sputter coater under vacuum (1 × 10^−4^ mbar). Transmission electron microscopy (TEM) micrographs were obtained using a FEI Tecnai G2 F20 electron microscope operated at 200 kV. Ammonia temperature programmed desorption (NH_3_-TPD) measurements were performed to determine the strength and concentration of acidic sites of the catalysts. NH_3_-TPD was performed on Micromeritics AutoChem II 2920 chemisorption analyser (~100 mg sample, 10°C min^−1^, He flow rate = 30 cm^3^ STP min^−1^). Fourier transform infrared transmission spectroscopy (FT-IR) was performed in a Bruker Vertex 70 spectrometer with the red-light emission from a Helium-Neon laser and the wide range MIR-FIR beam splitter and detector. The spectra were obtained at room temperature by 56 scans at 4 cm^−1^ resolution in the wavelength range of 400–1,200 cm^−1^.

### Catalysis

Before catalytic evaluation, all catalysts were ion-exchanged using 0.1 M aqueous NH_4_NO_3_ solution (1 g solids in 100 mL solution at 25°C under stirring). The 8 h process was repeated three times, and the resulting materials were washed using deionized water and dried at 110°C overnight in between the ion exchange treatments. Finally, the ion-exchanged samples were calcined in static air at 450°C for 5 h (heating rate = 5°C min^−1^, then cooled down naturally to room temperature). The parent Y was also calcined under the same condition to be converted to its H form before catalysis.

Catalytic cracking of 1,3,5-triisopropylbenzene (TiPBz) over the catalysts was performed at 325°C under atmospheric pressure using a pulse method (Zhai et al., 2003, 2006, 2008; Qi et al., 2015). The catalysts were pelletized (with ~250 mesh particle sizes), then loaded (~20 mg) in a borosilicate glass-tube liner (internal diameter, i.d. = 4 mm; outer diameter, o.d. = 6.3 mm; length = 72 mm, Restek). Deactivated glass wool (Restek) was used to hold the bed. Then, the tube was inserted into GC injector and heated to 325°C (from 50 to 325°C within 2 h). The catalyst was kept *in situ* for 1 h at 325°C before injections in order to remove moisture. Manual injection of 0.2 μL of TiPBz was performed using an Agilant SGE syringe (Trajan, 0.5BNR-5BV/0.63) with helium (He) as carrier gas. Reactants/products from the cracking reaction were analyzed inline by the GC (Varian 3400) equipped with a flame ionization detector (FID). Details of the GC method used are presented in Table S1. The analysis time for each injection was ~30 min and 22 total injections were performed (about 11 h).

Catalytic aldol condensation of benzaldehyde with 1-heptanol was carried out using Schlaker reaction tubes (Aldrich®) under N_2_ atmosphere. All the catalysts were dried before the catalytic tests in an oven at 180°C overnight to remove the moisture. In aldol condensation, the catalyst (200 mg) was first loaded into a 25 ml Schlaker tube followed by the addition of benzaldehyde (5 ml, 48.7 mmol), heptanal (1.2 ml, 8.7 mmol), and dodecane (0.2 ml, 0.87 mmol, as internal standard). Then, the resulting suspension was heated to 130°C (in an oil bath) under continuous stirring (of 300 rpm) and N_2_. The reaction mixture of about 0.2 ml was periodically sampled (diluted with ethanol and filtered) for GC analysis (Agilant 7820A with Agilent J&W HP-5 capillary column). Details of the GC method used for condensation reaction is presented in Table S2. Product identification was described elsewhere (Zhang et al., 2019).

## Results and Discussion

### Characterization of Y Zeolite, MZMs, and MCM-41

**Figure 1:**
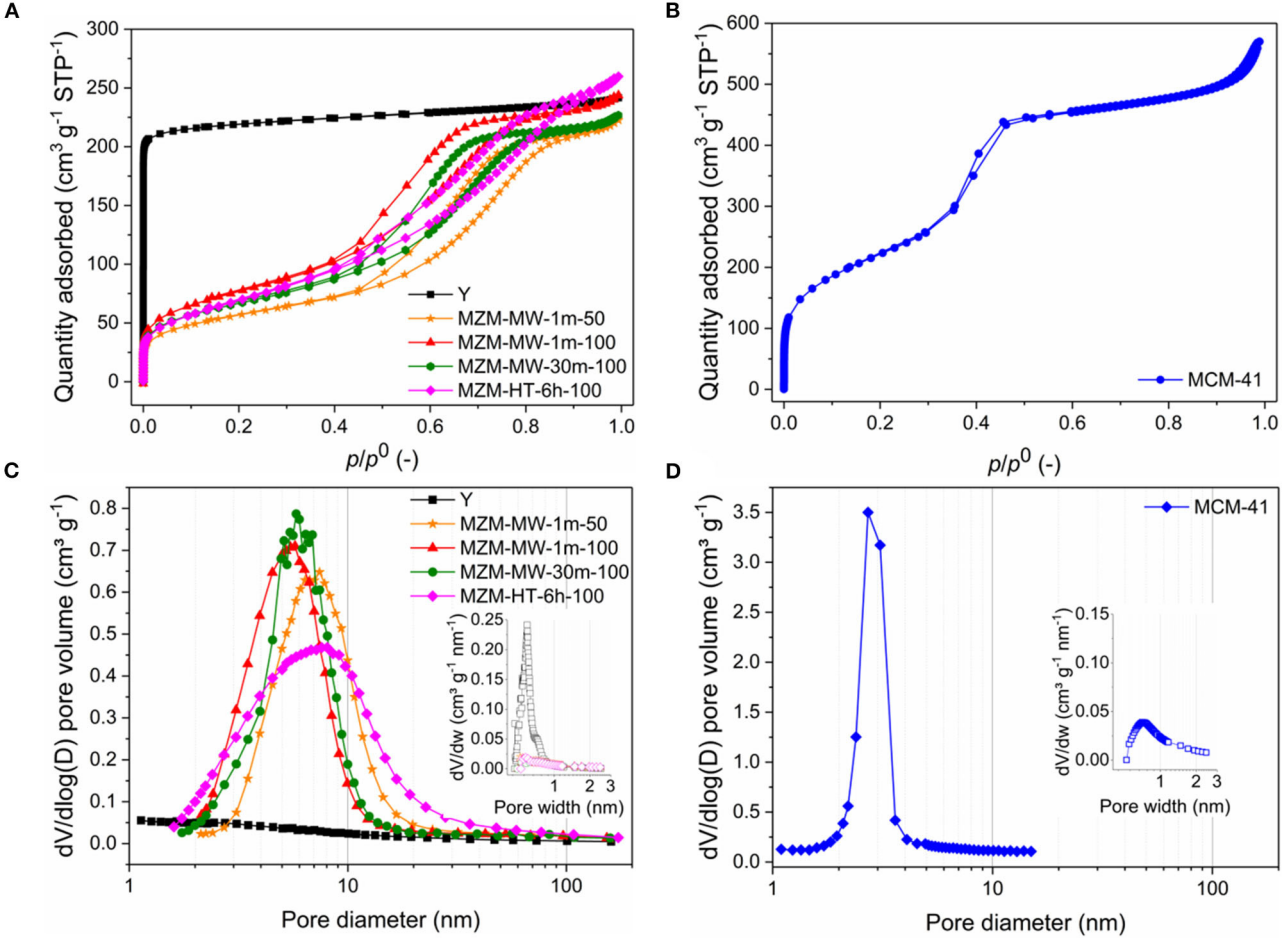
N_2_ adsorption-desorption isotherms of **(A)** MZMs (using Y zeolite as the reference) **(B)** MCM-41 and PSDs by the Barrett-Joyner-Halenda (BJH) method for **(C)** MZMs (using Y zeolite as the reference) and **(D)** MCM-41 [insets: the relevant micropore PSDs by the Horváth-Kawazoe (H-K) method].

The MWAC method is very effective. Regardless of whether it was at 50 or 100°C, 1 min treatment was sufficient to produce MZMs, which is confirmed by N_2_ adsorption-desorption analysis of the relevant materials (Figure 1A and Table 1). The parent Y zeolite displays a characteristic type I isotherm for microporous materials (Gregg et al., 1967; Awala et al., 2015). Conversely, the resulting MZMs show the type IV isotherms with H2 hysteresis loops according to the IUPAC classification (Sing et al., 1985), suggesting the well-developed mesoporous structure (Kresge et al., 1992; Qiao et al., 2014). MZMs were compared with MCM-41 for a detailed analysis of its physical and chemical properties. The isotherm of MCM-41 (Figure 1B) shows a steep increase of the adsorbed quantity at *P/P*° of ~0.38 [type A (H1) hysteresis] due to the capillary condensation of N_2_ in its mesopores. After that, the adsorbed quantity was less significant, confirming the cylindrical type pores of MCM-41 with a narrow distribution of uniform pores (Zhao et al., 2001). Comparatively, the isotherms of MZMs show relatively flat adsorption isotherms with moderately steep desorption curves over the range of *P/P*^0^, suggesting the presence of disordered structure of mesopores (i.e., wide pore size distribution) with interconnected networks. This was confirmed by the corresponding pore size distribution (PSD) of MZMs and MCM-41 obtained by the BJH method based on the adsorption branches, as shown in Figures 1C,D. This is in good agreement with the PSD of MZMs showing a wide distribution of mesopores (~2–10 nm) centered at about 5 nm, while MCM-41 showed a much narrower distribution centered at about 2.2 nm. Regarding the PSD of micropores in the materials (insets in Figures 1C,D), in comparison with the microporous Y zeolite, MZMs and MCM-41 show no presence of micropores, confirming the successful preparation of MZMs with the pure mesoporous structure. Table 1 summarizes the textural properties of the materials. Due to the relatively small mesopores in MCM-41, MCM-41 possesses a high BET surface area of 814 m^2^ g^−1^, being much higher than that of MZMs at <300 m^2^ g^−1^, which is in good agreement with the relevant adsorption capacity of the two mesoporous materials. MZMs have total specific pore volumes (*V*_total_) of >0.35 cm^3^ g^−1^ which is comparable to that of the parent Y zeolite.

**Table 1:** Textural properties and silicon-to-aluminum ratio (SAR) of materials.

**Material**	**Specific surface areas and pore volumes**						**SAR** **(–)**
	***S*_**BET**_** **(m^**2**^ g^**−1**^)**	**$V_{total}^{a}$** **(cm^**3**^ g^**−1**^)**	**$V_{micro}^{b}$** **(cm^**3**^ g^**−1**^)**	**$V_{meso}^{c}$** **(cm^**3**^ g^**−1**^)**	**$S_{micro}^{b}$** **(m^**2**^ g^**−1**^)**	**$S_{meso}^{b}$** **(m^**2**^ g^**−1**^)**	
Y	817	0.35	0.32	0.03	782	35	3.4
MZM-MW-1m-50	210	0.35	0.01	0.34	31	179	4.8
MZM-MW-1m-100	280	0.38	0.01	0.37	19	261	12
MZM-MW-30 m-100	236	0.35	0.01	0.34	18	218	10.7
MZM-HT-6h-100	242	0.4	0	0.4	0	242	13.0
MCM-41	814	0.88	0	0.88	0	814	−

a *Total volume adsorbed at p/p^0^ = 0.99*.

b *Based on the t-plot method*.

c *V_meso_ = V_total_ – V_micro_*.

Considering the post-synthetic method used for preparing MZMs, both the MWAC and conventional hydrothermal treatment can produce MZMs with comparable porous properties. However, the MWAC method can intensify the complexation reaction between EDTA and Al species in the parent zeolite Y significantly compared to the conventional HT treatment. MWAC method can produce MZMs at a comparatively mild temperature of 50°C with the considerably reduced treatment time of 1 min, whereas the conventional HT method requires 6 h post-treatment at 100°C. By comparing the properties of MZMs produced under different conditions (i.e., by varying the treatment time and temperature), MWAC method showed insignificant dependence on the treatment time and temperature, suggesting that (i) the rapid volumetric heating due to MW irradiation was less influential on the extraction of Al species and (ii) the slow hydrolysis of framework Al in the zeolite Y might be skipped.

The excellent performance of the MWAC method can be assigned to the following reasons: (i) the thermal dispersion of the chelating agent EDTA in the zeolite framework was improved by microwave irradiation (Feng et al., 2012)—this might be attributed to the intensified interaction between Al species in the zeolite framework and the chelator leading to the improved diffusion of chelator molecules into zeolite pores (Chandra Shekara et al., 2011); (ii) the good microwave absorption property of the framework Al compared with the framework Si (González et al., 2011), accelerating the selective interaction between the framework Al with the chelator; and (iii) the relatively low bond energy of the Al-O in comparison with that of the Si-O (Smith and Bailey, 1963; Muraoka et al., 2016). Accordingly, the interaction between the framework Al and EDTA was more intensive under microwave irradiation than under the conventional hydrothermal condition (González et al., 2011).

**Figure 2:**
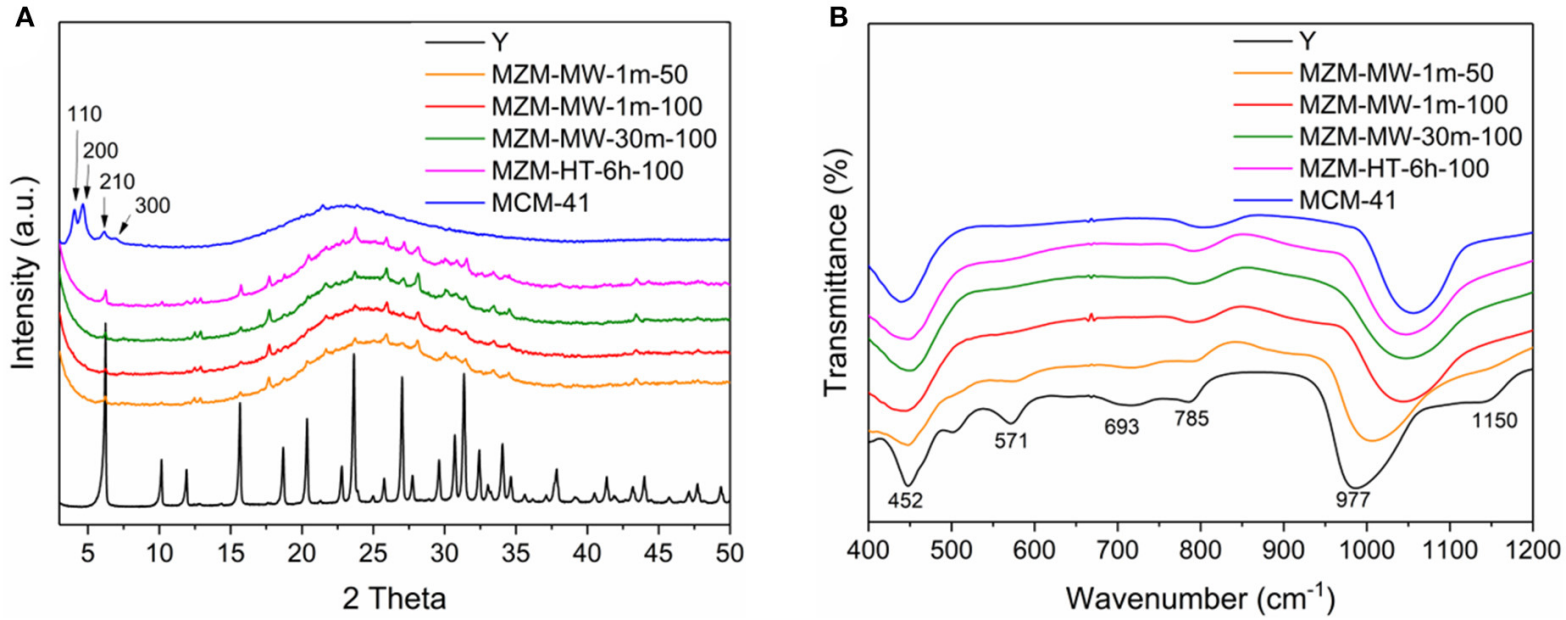
**(A)** XRD patterns and **(B)** FT-IR spectra of the Y zeolite, MZMs, and MCM-41.

Findings by XRF analysis (as shown in Table 1) show that the bulk SAR values of the materials was ~3.4 for the parent Y zeolite, while that for MZMs varies depending on the condition used, that is, the relatively low temperature seems preserve the acidity in comparison with the higher one (SAR = ~4.7 for MZM-MW-1m-50, and about 11 for MZM-MW-1m-100 MZM-MW-30m-100, respectively). The findings from XRF analysis show that SAR values of the resulting MZMs could be preserved to some extent, and thus the MZMs still have acidity. XRD patterns of MZMs (as shown in Figure 2A) show that the MZMs are largely amorphous with the typical broad signal corresponding to silica at around 25° 2θ. However, the crystalline phase of the parent Y is still preserved to a very small extent, as evidenced by the remaining Y zeolite (111) (2θ = 6.2°), (331) (2θ = 15.6°), and (533) (2θ = 23.8°) planes in MZMs (Aghakhani et al., 2014; Choo et al., 2019).

For the pure silica MCM-41, its XRD diffraction pattern shows the highly amorphous silica together with the well-resolved low-angle diffraction peaks indexed as (110), (200), (210), and (330), suggesting its hexagonally-ordered structure (Corma et al., 1996; Kruk et al., 1999). FTIR spectra of the materials (Figure 2B) show the common vibration peaks at about 452, 785, and 977 cm^−1^. The bands at ~452 cm^−1^ are assigned to internal tetrahedral vibration of TO_4_ (where T = Si or Al). The bands at ~785 cm^−1^ are assigned to external linkage internal tetrahedral symmetrical stretching (Liu et al., 2013). Compared with the parent Y zeolite, the absence of the bands at ~693 cm^−1^ (the external linkage symmetrical stretching) and the lower intensity of the bands at ~785 cm^−1^ for the mesoporous materials might be due to the their relatively small particle sizes (Don et al., 2016). The bands at ~977 cm^−1^ are attributed to the internal tetrahedral asymmetrical stretching. Compared with the parent Y zeolite, the IR peak at ~977 cm^−1^ for MZMs, as well as MCM-41, shift to a higher wavenumber with lower intensity due to the absence of Al–O bond (with the bond length of 1.75 Å) in MZMs and MCM-41 (the Si–O bond length = 1.61 Å). The strong electronegativity of Si leads to a relevantly high vibration frequency, causing the band's shifts to higher wavenumbers in the mesoporous materials. Parent Y zeolite also shows the IR peak at about 570 cm^−1^, being assigned to the double six-member-ring (Tan et al., 2007). The absence of this IR band in MZMs suggests the mesoporous nature of MZMs. The standard MWAC treatment of zeolites is at 100°C. Based on the findings above, i.e., the comparable properties of MZMs, especially the textural property, MZM-MW-1m-100 was chosen as the model MZM to perform the comparative characterization and catalysis. Additionally, 1 min treatment time also indicates the significant reduction in the energy consumption.

**Figure 3:**
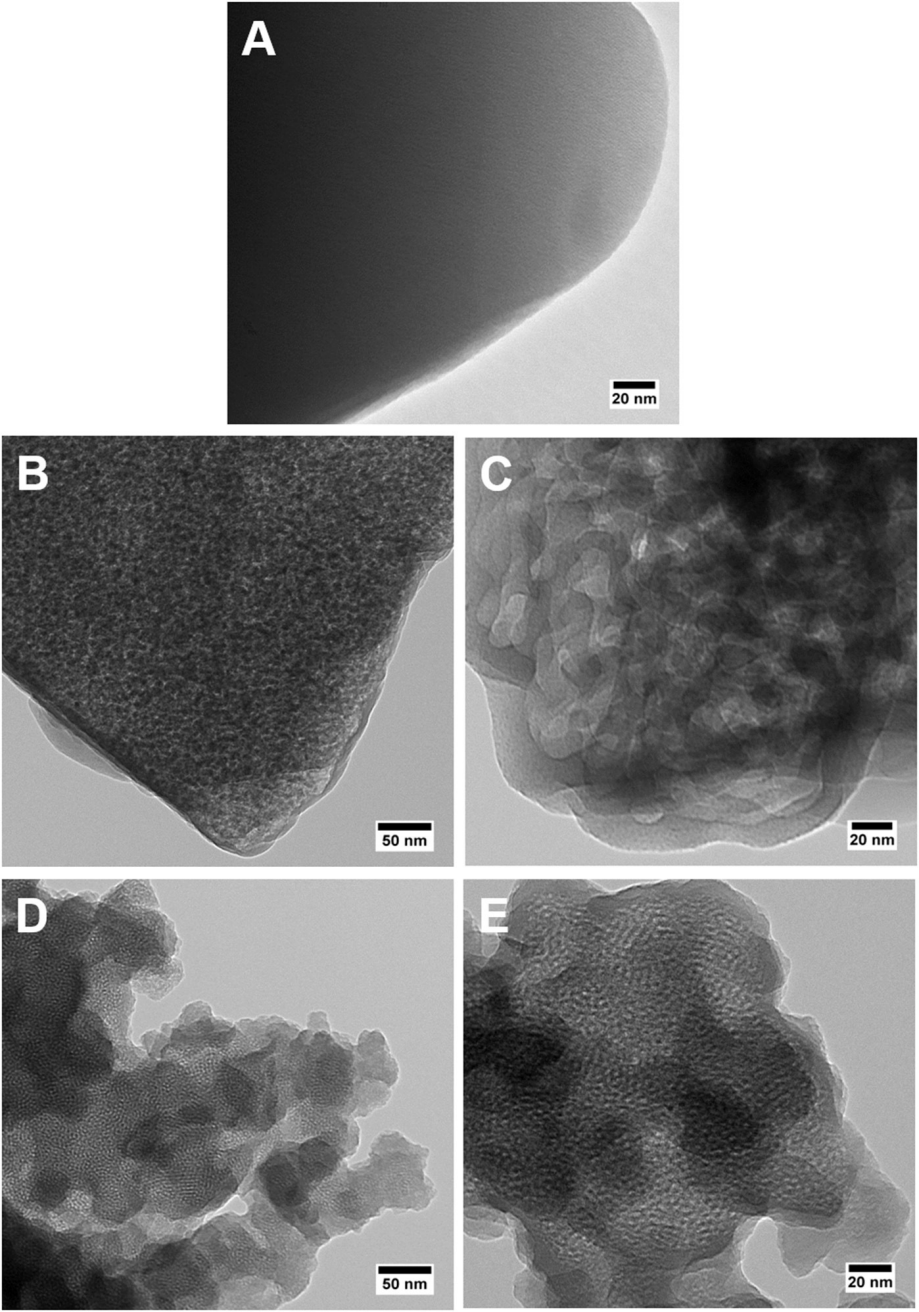
TEM micrographs of **(A)** zeolite Y, **(B,C)** MZM-MW-1m-100, and **(D,E)** MCM-41.

**Figure 4:**
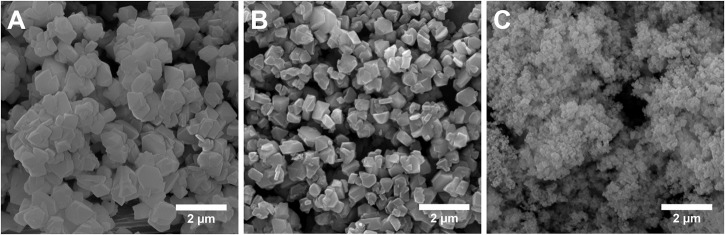
SEM micrographs of **(A)** zeolite Y, **(B)** MZM-MW-1m-100, and **(C)** MCM-41.

TEM image in Figure 3A shows a dense and uniform phase of the parent Y zeolite (the starting material for making MZMs), suggesting the absence of mesoporosity. As shown in Figures 3B,C, TEM analysis of MZM-MW-1m-100 reveals the mesoporous structure with randomly arranged mesopores. Compared to MCM-41 (TEM images in Figures 3D,E), which has ordered one-dimensional hexagonal mesoporous structure, the pore sizes of MZMs are much larger, being in line with the results by N_2_ physisorption analysis. SEM analysis of the materials (Figure 4) shows that the parent Y zeolite (Figure 4A) and MZM (Figure 4B) have the similar morphology with the particle sizes <1 μm, but the crystals of parent Y are aggregated, as well as being larger than MZMs. The particles of MCM-41 (Figure 4C) are mostly agglomerated and much smaller than that of the parent Y zeolite and MZM. Relevant characterization data of MZM-HT-6h-100 are shown in Figures S1–S3 and Table S3, which are not discussed here since its property is comparable to that of MZMs prepared by the MWAC method.

**Figure 5:**
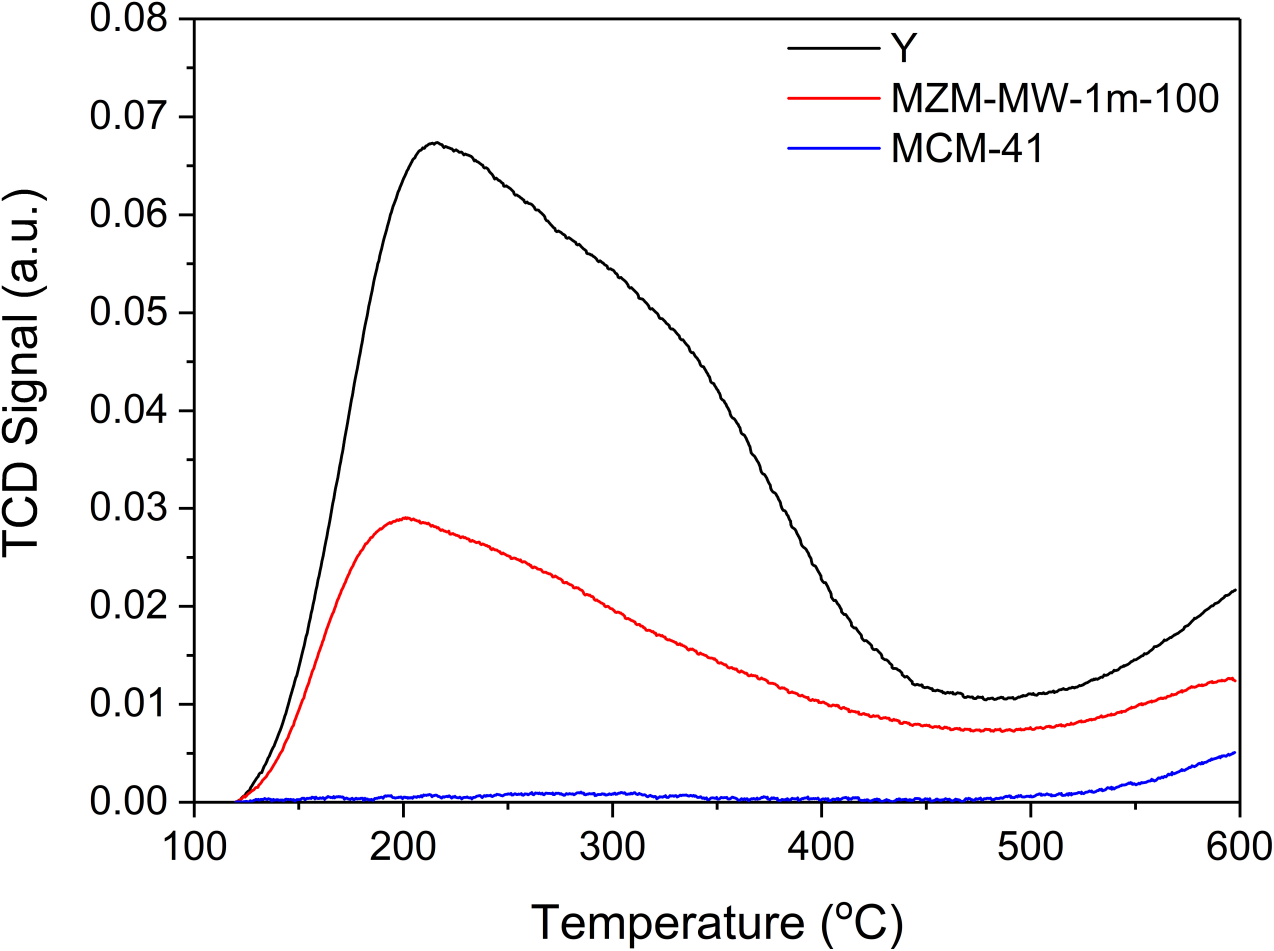
NH_3_-TPD profiles of the parent zeolite Y, MZM-MW-1m-100, and MCM-41.

The acidic properties of the materials under investigation were probed by NH_3_-TPD, and the relevant results are presented in Figure 5 and Table 2. MCM-41 barely shows any acidity due to the absence of Al species in its framework, whereas the parent Y and MZM show TCD signals due to desorption of the adsorbed NH_3_ on their acidic sites. NH_3_-TPD curves can be deconvoluted into two desorption peaks appearing at about 200 and 300°C, corresponding to their weak and strong acid sites, respectively. The MWAC method can dealuminate the zeolite to reduce its acidity. The low temperature peak at ~200°C may be due to the desorption of NH_3_ adsorbed on the weak Lewis acid sites, that is, the exposed Al^3+^ cations without the Al-O-Si bridges, while the high temperature peak at ~300°C corresponds to ammonia desorption from the strong Brønsted acid sites, i.e., the surface hydroxyls due to the remaining Al-O-Si bridges in the zeolitic framework (Qin et al., 2011; Wang et al., 2015; Zhang et al., 2019). Compared to the siliceous MCM-41, the developed MZM possesses significantly more acidity (as shown in Table 2), which can be beneficial to catalysis.

**Table 2:** Analysis of NH_3_-TPD of the catalysts.

**Sample**	**Temperature at maximum (**^****°****^**C)**		**Weak acidity^**a**^** **(mmol g^**−1**^)**	**Strong acidity^**b**^** **(mmol g^**−1**^)**	**Total acidity** **(mmol g^**−1**^)**
	**First peak**	**Second peak**			
Y	209	320	1.053	0.819	1.872
MZ-MW-1m-100	193	288	0.358	0.254	0.612
MCM-41	0	276	0	0.006	0.006

a *First peak*.

b *Second peak*.

### Catalytic Cracking of 1,3,5-Triisopropylbenzene (TiPBz) Over the Catalysts

**Figure 6:**
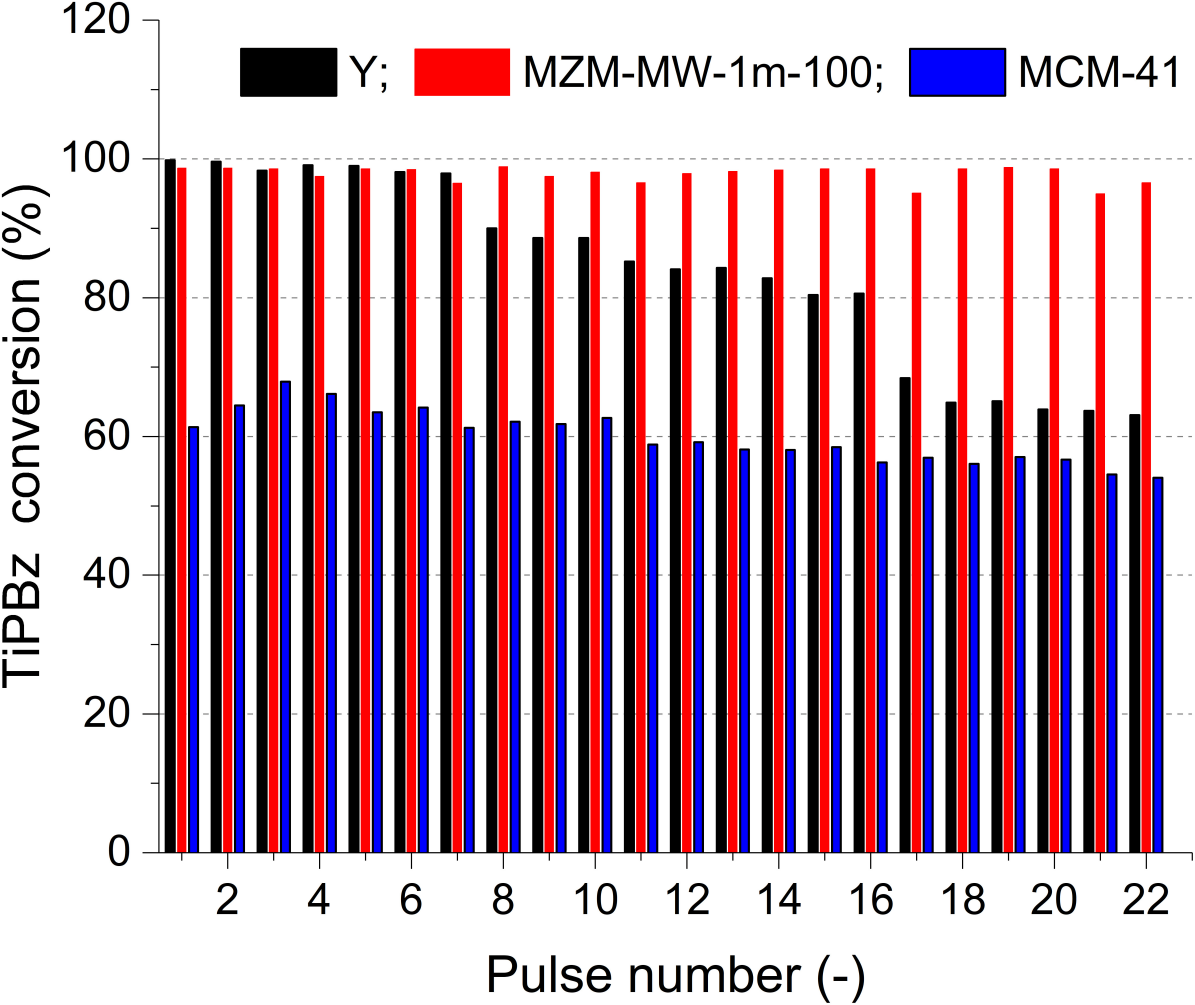
Conversion of TiPBz over the parent Y zeolite, MZM-MW-1m-100, and MCM-41 as a function of pulse number.

Aluminosilicate and silica mesoporous materials are potentially beneficial to the petrochemical conversion processes, especially the important fluidized catalytic cracking (FCC). Hence, the catalytic cracking activity of MZMs was evaluated in reference to the parent Y zeolite and MCM-41 using a pulse method and 1,3,5-triisopropylbenzene (TiPBz, as the model compound). Figure 6 shows the activity (regarding the absolute conversion of TiPBz) of the materials as the function of pulse number. The parent Y zeolite showed the initial good TiPBz conversions of ~98%, which rapidly deactivated to ~62% after 22 injections. This result suggests fast coke deposition on the external surface of Y crystals. TiPBz has a kinetic diameter of 0.94 nm (Jiao et al., 2020), being larger than the intrinsic pore width of Y zeolite (i.e., 0.74 nm). Thus, the accessibility issue might enable the initial dealkylation reaction of bulky TiPBz on the external surface of zeolite Y crystals, causing the rapid coke deposition and deactivation. MZM and MCM-41 showed rather stable catalytic performance thanks to their mesoporous structures. Although the silica-based MCM-41 only has insignificant acidity, it demonstrated a fairly good ability to crack TiPBz with conversions at 60 ± 3%. Comparably, the aluminosilicate MZMs showed much better performance in cracking TiPBz than MCM-41 with conversions at 98 ± 1% during the test. The excellent catalytic performance of the MZM-MW-1m-100 can be explained by (i) the relatively large pore sizes (2–10 nm for MZMs vs. 2.2 nm for MCM-41) and (ii) the presence of acidity in the mesoporous framework.

**Figure 7:**
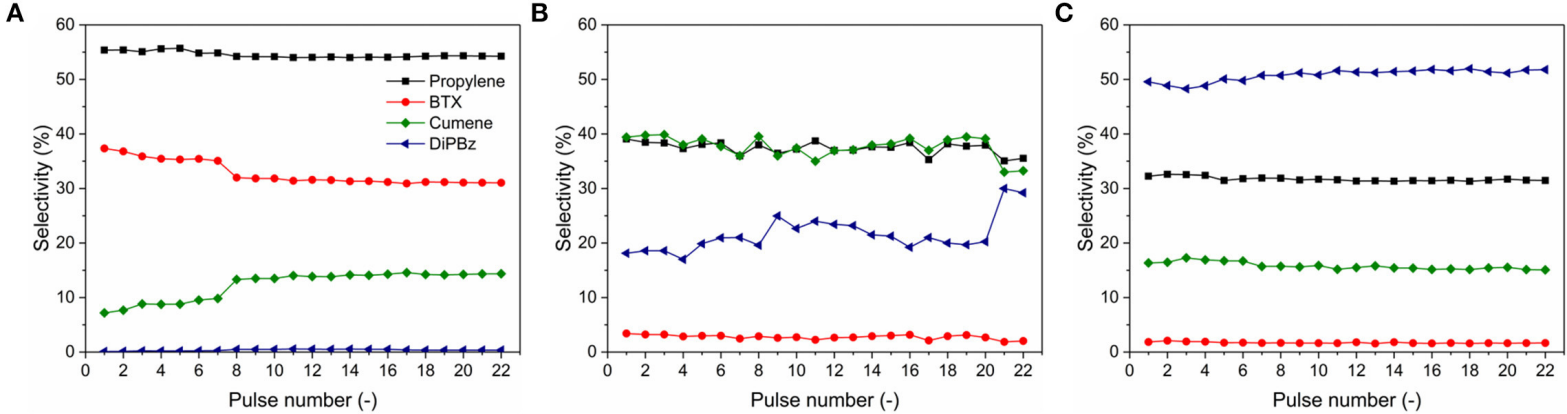
Product selectivity of catalytic TiPBz cracking over **(A)** Y zeolite, **(B)** MZ-MW-1m-AT, and **(C)** MCM-41.

The relevant selectivity to different products is shown in Figure 7. Y zeolite showed relatively high selectivity to propylene (at 54.5 ± 0.5%) and benzene, toluene, and xylene isomers (BTX, at 32 ± 2%), low selectivity to cumene (at 12 ± 3%) and insignificant selectivity to 1,3- and 1,4-diisopropylbenzene (DiPBz, at 0.37 ± 0.14%), as shown in Figure 7A. The results suggest a fairly complete cracking of TiPBz, proving the successive cracking of DiPBz (kinetic diameter of 0.84 and 0.73 nm for 1,3- and 1,4-DiPBz, respectively) and cumene (kinetic diameter of 0.68 nm) within the microporous framework, hence the high selectivity to propylene and BTX. In Figure 7B, one can see that the main products of cracking reactions over MZM-MW-1m-100 were propylene and cumene with the selectivity at 38 ± 2 and 38 ± 2%, respectively. The selectivity to DiPBz was 21.5 ± 3%, whereas the selectivity to BTX was relatively low at 3 ± 0.5%. Conversely, over MCM-41, DiPBz (Figure 7C), propylene and cumene were the main products with the relevant selectivity of 50 ± 1, 32 ± 1, and 16 ± 0.5%, respectively, whereas the selectivity to BTX was insignificant (i.e., 1.7 ± 0.1%). The catalytic performance of MZM-HT-6h-100 are shown in Figure S4, being comparable to that of MZM-MW-1m-100.

The variation of selectivities in the cracking catalysis over different catalysts under investigation can be mainly due to their porous properties. The parent Y only possesses the intrinsic micropores with the pore width of 0.74 nm, whilst the reactant of TiPBz has a kinetic diameter (KD) of 0.94 nm (Funke et al., 1997; Jiao et al., 2020). Therefore, the initial cracking of TiPBz over the parent Y might only proceed on the outer surface of the parent Y crystal. The cleavage of isopropyl groups of TiPBz produces propylene (KD = 0.45 nm) (Baker, 2012), *para*-/*meta*-DiPBz (KD = 0.71) (Funke et al., 1997) and cumene (KD = 0.68 nm) (Jahandar Lashaki et al., 2012; Jiao et al., 2020), respectively. Considering the KD of DiPBz and cumene, it is likely that they can diffuse into the framework of the parent Y, hence enabling the further cracking reactions to cumene (from *para*-/*meta*-DiPBz) and BTX (KD of benzene and toluene = 0.59 nm, KD of xylene isomers = 0.58–0.68 nm Baertsch et al., 1996; Funke et al., 1997, from *para*-/*meta*-DiPBz and cumene. Although the intrinsic micropore of Y zeolite promoted the size selectivity, deactivation was measured, which might result from the carbon deposition within the microporous framework, as evidenced by the reduced TiPBz conversion (Figure 6) and selectivity to BTX (Figure 7A), as well as by the thermogravimetric analysis (TGA) of the used catalysts (Figure S5). The selectivity of the cracking catalysis over the two mesoporous materials can also be correlated with their pore structures. The MZM catalyst was derived from Y zeolite with a wide PSD of 2–10 nm, which allows the unrestricted molecular transport of reactant/products, explaining the stable high conversion of TiPBz. However, due to the absence of the intrinsic micropores, cracking reactions over MZM-MW-1m-100 were selective to the relatively large products of cumene and DiPBz (compared to BTX). MCM-41 has a 1-dimensional pore system with the regular arrangement of cylindrical mesopores at 2.2 nm, being larger than the KDs of the reactant and products. The catalysis over MCM-41 was selective to DiPBz (Figure 7C), which was due to the insignificant acidity of MCM-41, and the reaction was halted at DiPBz without further cracking (i.e., insignificant selectivity to BTX). The results of catalytic cracking over the three materials under investigation are interesting, demonstrating the trade-offs between accessibility and deactivation, and between the activity and shape selectivity.

### Catalytic Aldol Condensation Over the Catalysts

**Figure 8:**
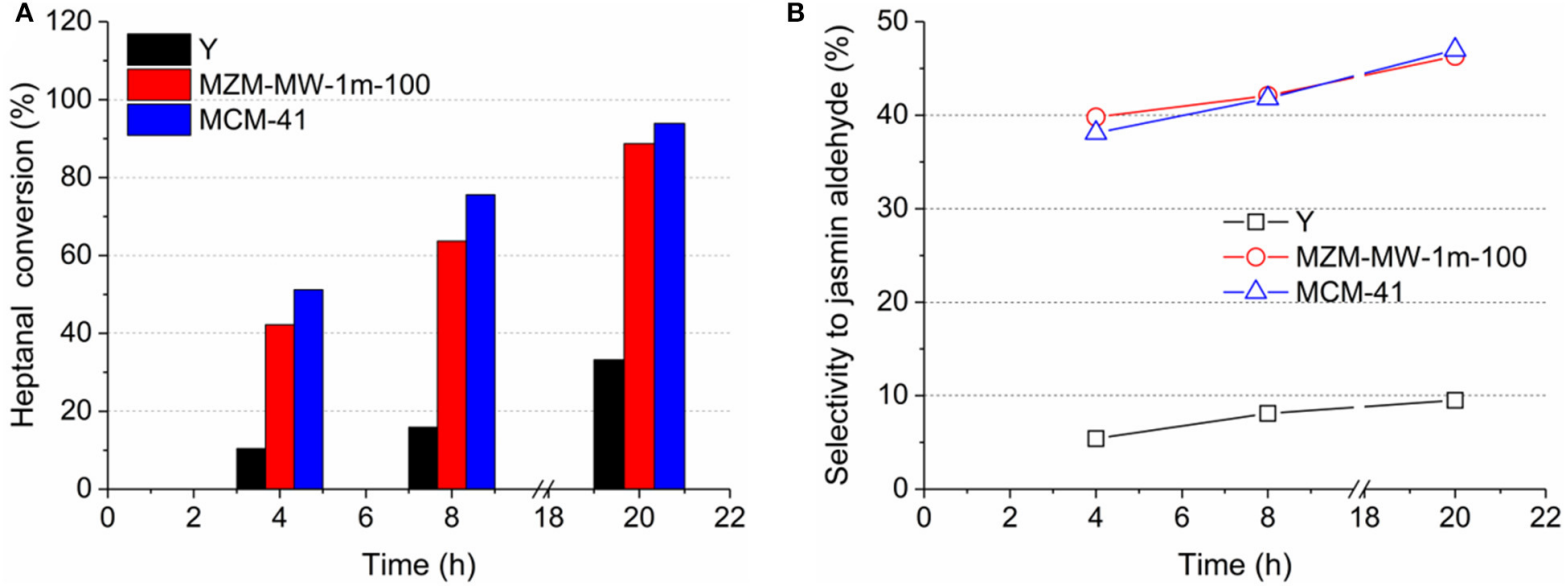
**(A)** conversion of 1-heptanal and **(B)** selectivity to jasmin aldehyde as a function of reaction time in aldol condensation over the parent Y zeolite, MZM-MW-1m-100, and MCM-41.

To probe the effectiveness of mesopores, the catalytic aldol condensation in the liquid phase was carried out over the materials under investigation. Aldol condensation of benzaldehyde with 1-heptanal is less relevant with the strong acidity of the catalyst (Zhang et al., 2019), and can be catalyzed by the surface silanol groups (Jentys et al., 1996, 1999; Xu et al., 2002). Sufficient space in the framework catalyst is required to allow the formation of the bulky product of jasmin aldehyde. Accordingly, mesoporous materials can be beneficial to the selective formation of jasmin aldehyde. As shown in Figure 8A, the parent Y displayed the lowest activity in terms of 1-heptanal conversion at the end of the reaction (i.e., about 32% at 20 h). In contrast, the mesoporous MZM-MW-1m-100 and MCM-41 exhibited improved activity (1-heptanal conversion at 20 h: about 90% for MZ-MW-1m-100 and 94% for MCM-41) compared with the parent Y zeolite. The selectivity to jasmin aldehyde was only ca. 9% at the end of the reaction over the microporous parent Y, owing to the pore size limitation (intrinsic micropore diameter of Y zeolite = 0.74 nm). Both MZM-MW-1m-100 and MCM-41 showed the relatively good selectivity to jasmin aldehyde, and they are comparable, i.e., about 47% at 20 h as shown in Figure 8B. Since both MZM-MW-1m-100 and MCM-41 are mesoporous, the promoted formation of jasmin aldehyde in the system can be expected.

### Assessment of Siliceous and Aluminosilicate Mesoporous Materials

In order to assess the developed MZMs preliminarily, the comparison in reference to some state-of-the-art siliceous and aluminosilicate mesoporous materials with the emphasis on their preparation methods, properties, and health, safety, and environment (HSE) aspects, as well as the preliminary economic analysis, were made, as shown in Table S4. Generally, MZMs, especially ones prepared by the MWAC method facilitated by microwave irradiation, outperform other reference materials comparatively in some aspects. The key advantages of MZMs by the MWAC method can be identified, such as the relative high-acidity, low-toxicity (concerning the chemicals used during their preparation), time- and energy-efficiency, and low-cost (N.B. the preliminary analysis did not include the relevant energy consumption for making the parent Y zeolite used for the post-treatment). Importantly, without the necessity of using mesoscale templates, such as surfactants and polymers in the preparation of MZMs, MZMs can be sustainable and eco-friendly, i.e., without the need for removing the templates via calcination during preparation, which usually requires high temperatures at >450°C to burn the expensive templates and release toxic flue gases. Additionally, the preliminary cost analysis was made for MZMs prepared by the MWAC and conventional hydrothermal treatments based on the laboratory-scale preparation of 1 g MZM in reference to MCM-41 (using the retail price of the commercial siliceous MCM-41 from Sigma-Aldrich®). The economic estimation was based on the consideration of the costs of raw materials, chemical used, and energy consumption (the details of the relevant calculations are presented in the Supplementary Material). Accordingly, based on the chemicals, materials and methods used in this work, the production of 1 gram of MZMs using the MWAC method costs about £0.96, while the relevant cost for making 1 g MZM using the conventional hydrothermal method (both at 100°C) is about £1.6. Since the costs of the starting material and chemicals and energy for the workup are same for the two methods, the key difference between them is the cost related to the energy consumption during the post-synthetic treatments. Based on the laboratory practice of the chemical treatment, the real electricity usage of the methods was measured using a plug power meter. The hydrothermal method used 1.05 kWh, being 21 times as high as that of the MWAC method (i.e., 0.05 kWh). Using the commercial MCM-41 as the reference, 1 g costs £18.5 (retail price), and is much more expensive than the cost estimated for the MZM. Although the state-of-the-art mesoporous materials (Table S4) possess the high level of mesoporosity, their current syntheses may be less sustainable and eco-friendly than the microwave-facilitated MWAC method for making MZMs, resulting in a great challenge for their practical applications on large scales. All of the aforementioned advantages may help to make MZMs (prepared by the MWAC method) relatively eco-friendly and cost-effective, hence showing the potential for further exploitation toward practical applications on a large scale.

## Conclusions

Mesostructured zeolitic materials (MZMs) were prepared using the post-synthetic chemical treatment of Y zeolite, being particularly effective under the microwave irradiation (known as the MWAC method). Using EDTA as the chelator, the MWAC method can produce MZMs with a short treatment time of 1 min at both 50 and 100°C. NH_3_-TPD measurement of MZMs showed the presence of acidities, resulting in the improved activity in catalytic cracking of TiPBz with a high yet stable conversion of ≥97% (over the course of the pulse experiments). Comparatively, the reference catalysts of MCM-41 and Y zeolite showed the low conversion, as well as deactivation, respectively. Using the aldol condensation to probe the effectiveness of the mesoporosity in MZMs, the results showed the comparable ability of MZMs and MCM-41, allowing the formation of the bulky product of jasmin aldehyde with the selectivity at ~47% (after 20 h), whereas the microporous Y only achieved the selectivity at ~9%. Based on the preliminary cost analysis of preparing MZMs on the laboratory scale, 1 gram of the MZMs about costs £0.96 (including the cost of the reagents used and the energy consumption) which is much cheaper than the retail price of MCM-41 (at about £18). Additionally, the comprehensive comparison of MZMs in reference to the current ordered/disordered mesoporous materials was performed, showing the possible advantages MZMs in the aspect of high acidity, time-/cost-effective and environmentally friendly preparation and sustainability.

## Data Availability Statement

All datasets generated for this study are included in the article/Supplementary Material.

## Author Contributions

XF and YJ: conceptualization of the research. SA, SX, RZ, YJ, and AG: synthesis and characterization of materials and catalysis. SA and XF: data analysis. XF: resources and project management, and revisions of the manuscript. SA: initial draft preparation and data interpretation/visualization. All authors contributed to the article and approved the submitted version.

## Conflict of Interest

XF and YJ are inventors and SA is the contributor on a patent application submitted by the UMI3 Limited (The University of Manchester's agent for Intellectual Property commercialization and technology transfer) based on the intellectual property of the work (International Publication Number: WO 2020/053592 A1). The remaining authors declare that the research was conducted in the absence of any commercial or financial relationships that could be construed as a potential conflict of interest.
