# Supplementary material for: Mesoporous Zeolitic Materials (MZMs) Derived From Zeolite Y Using a Microwave Method for Catalysis
Source: Front Chem. 2020 Jun 30;8:482. doi: 10.3389/fchem.2020.00482 (PMC7338609; doi:10.3389/fchem.2020.00482)
Supplement: Supplementary file 1 [file Table_1.DOCX]

**Mesoporous zeolitic materials (MZMs) derived from zeolite Y using a microwave method for catalysis**

## **Supporting Information (SI)**

**Table S1.** GC method used in the catalytic cracking of TiPBz.

| **Injection** | - Injection volume = 0.2 μl (manual injection) |
| --- | --- |
| **Inlets** | - Heater temperature = 325 °C - Split ratio = 200:1 - Pressure = 16 psig - Flow rate = 200 mL min^−1^ |
| **Column & Oven** | - Stabilwax® (0.32 mm i.d., 30 m length, 1 μm film thickness) - Carrier gas = He (at 200 mL min^−1^)   Temperature programme:   - Initial temperature = 80 °C (Oven) - Initial hold time at 80°C = 5 min - Heating rate = 10 °C min^−1^ - Final temperature = 220 °C - Final hold time at 220 °C = 10 min |
| **Detector** | - Detector type = Flame ionization detector (FID) - Detector temperature = 300 °C |

**Table S2**. GC method used in the aldol condensation for benzaldehyde, heptanal and dodecane (internal standard) using ethanol as a solvent.

| **Injection** | - 10 μl Agilent syringe - 1 μl injection volume with ×2 pre-injection and ×2 post-injection washes |
| --- | --- |
| **Inlets** | - heater temperature = 280 °C - split ratio = 50:1 (split flow = 65 ml min^−1^) |
| **Column** | - temperature limits = −60 °C to 325 °C - stationary phase = (5%-Phenyl)-methylpolysiloxane - dimension = 30 m (length)×0.32 mm (ID)×0.25 μm (film) - carrier hydrogen flow = 1 ml min^−1^ (20 cm sec^−1^) |
| **Oven** | - initial temperature = 130 °C - initial hold time at 130 °C = 2.5 min - ramp = 15 °C min^−1^. - final temperature = 240 °C - final hold time at 240°C = 5 min |
| **Detector** | - Flame ionization detector (FID) - heater temperature = 320 °C - air flow = 400 mL min^−1^ - H_2_ fuel flow = 30 ml min^−1^ - N_2_ make-up flow = 20 ml min^−1^ |
| **Signals** | - data rate = 100 Hz - minimum peak width = 0.002 min - zero at the start of the run |

| 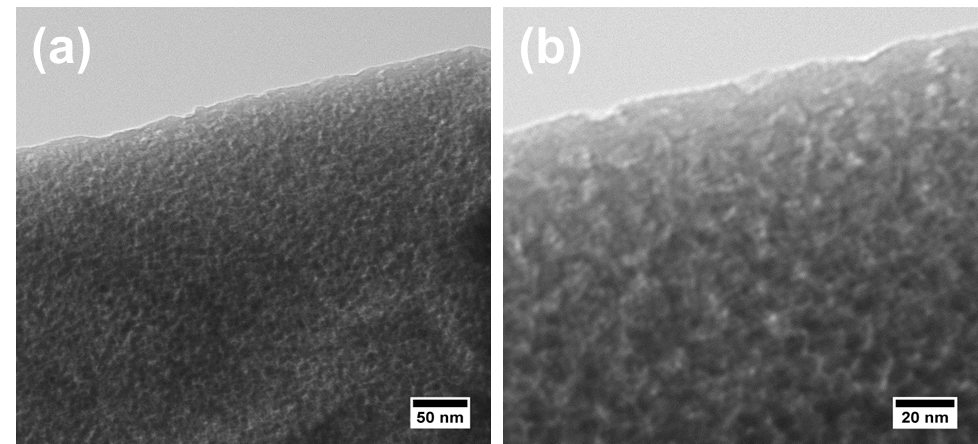 |
| --- |

**Figure S1.** TEM images of MZM-HT-6h-100 with different magnifications.

|  |
| --- |

**Figure S2.** SEM image of MZM-HT-6h-100.

|  |
| --- |

**Figure S3.** NH_3_-TPD profiles of MZM-HT-6h-100.

**Table S3**. Acidic properties of MZM-HT-6h-100.

| Sample | Temperature at maximum  [°C] | | | | Weak acidity^a^  [mmol g^−1^] | | Strong acidity^b^  [mmol g^−1^] | | Total acidity  [mmol g^−1^] | |  |
| --- | --- | --- | --- | --- | --- | --- | --- | --- | --- | --- | --- |
|  | First peak | | Second peak | |  |  |  |  |  |  |  |
| MZM-HT-6h-100 | | 208.9 | | 322.4 | | 0.288 | | 0.216 | | 0.504 | |

^a^first peak; ^b^second peak

| 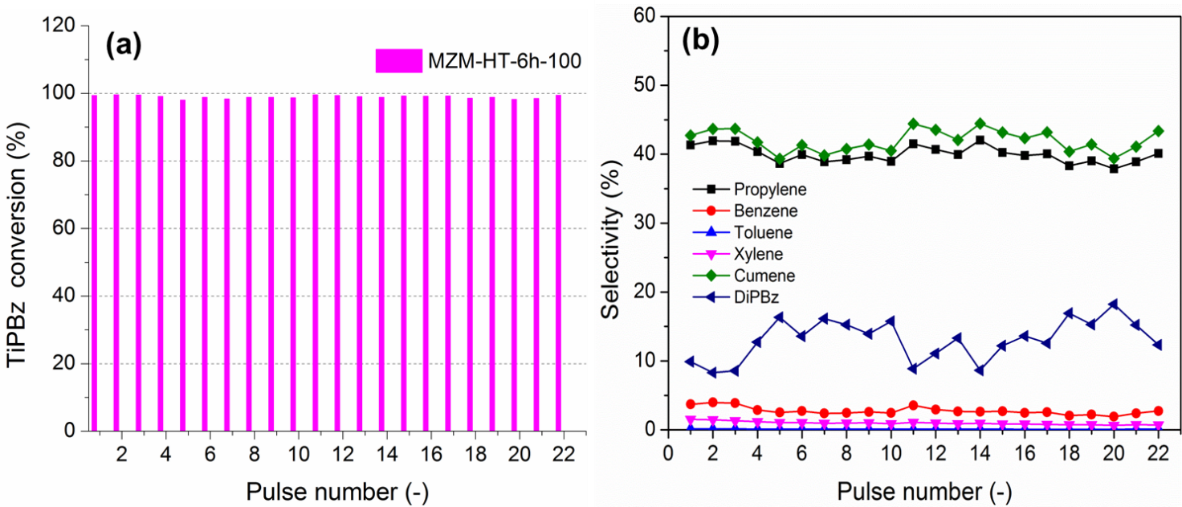 |
| --- |

**Figure S4.** (a) Conversion of TiPBz and (b) products selectivity over MZM-HT-6h-100 as a function of pulse number.

|  |
| --- |

**Figure S5**. TG curves of the used MZMs, parent Y zeolite and MCM-41 from the pulse experiments in catalytic cracking of TiPBz at 325 °C.

**Table S4.** Preliminary comparison of the state-of-the-art mesoporous ordered and discorded siliceous and aluminosilicate materials.

| Materials | Preparation method | Precursors/templates | Advantages and Disadvantages | | | | | | | | | Ref. | |
| --- | --- | --- | --- | --- | --- | --- | --- | --- | --- | --- | --- | --- | --- |
|  |  |  | Level of mesoporosity | Acidity | Toxicity | Time | Energy consumption | | Comments on cost | | |  |  |
| **Silica-based materials** | | | | | | | | | | | | | |
| MCM-41 | HS | - Si sources - CTAB as template | **High**  Uniform pore shape,  1.8–10 nm | **Very** **low** | **High**  due to the use of templates | **High**   - 48 h synthesis - 6 h calcination to remove template | | **High**   - Synthesis at 150 ºC, - Calcination at 540 ºC | | **High**   - Expensive (templates) - High energy consumption due to length synthesis | | | ([Beck et al., 1992](#_ENREF_1)) |
| SBA-15 | HS | - Si sources - HCl - P123, DMF and CTAB as templates | **High**  Uniform pore shape,  5–30 nm | **Very** **low** | **High**  due to the use of templates | **High**   - 44 h synthesis - 6 h calcination to remove template | | **High**   - Sequential synthesis at 40 and 100 ºC - Calcination at 550 ºC | | **High**   - Expensive (templates) - High energy consumption due to length synthesis | | | ([Zhao et al., 2000](#_ENREF_9)) |
| **Aluminosilicate materials** | | | | | | | | | | | | | |
| MAS-5 | HS | - Si and Al sources - TEAOH - CTAB | **High**  Uniform pore size at  2.7 nm | **High** | **High**  due to the use of templates | **High**   - Several hours to prepare the solution followed by 48 h synthesis - 6 h calcination to remove template | **High**   - Synthesis at 100–150 ºC - Calcination at 550 ºC | | **High**   - Expensive (templates) - High energy consumption due to length synthesis | | ([Zhang et al., 2001](#_ENREF_7)) | | |
| MAS-7 | HS | - Si and Al sources - NaOH - TEAOH - P123 - HCl | **N/A**  uniform pore size at  7.6 nm | **High** | **High**  due to the use of templates | **High**   - Multiple synthesis steps at 4, 20 and 24 h. - 6 h calcination to remove template | **High**   - Synthesis at 140, 40 and 100 ºC - Calcination at 550 ºC | | **High**   - Expensive (templates) - High energy consumption due to length synthesis | | ([Han et al., 2001](#_ENREF_2)) | | |
| DMAS | HS | - Si and Al sources - NaOH - CTAB | **Medium**  uniform pore size at  2.9 nm | **High** | **High**  due to the use of templates | **High**   - 72 h preparation - 72 h synthesis - 8 h calcination | **High**   - 25 ºC - N/A (certain temperature) - Calcination at 550 ºC | | **High**   - Expensive (templates) - High energy consumption due to length synthesis | | ([Zhao et al., 2001](#_ENREF_8)) | | |
| MMZ_beta_  MMZ_zsm-5_ | Top-down followed by bottom-up | - H-beta zeolite - H-ZSM5 zeolite - NaOH - CTAB - HCl/ethanol for washing | **High**  uniform pore shape at 2.8 nm | **High** | **High**  due to the use of templates | **High**   - 24 h preparation - 24 h synthesis - 3 h calcination | **High**   - Synthesis at 25 and 100 ºC - Calcination at 550 ºC | | **High**   - Expensive (templates) - High energy consumption due to length synthesis | | ([Lee et al., 2008](#_ENREF_3)) | | |
| Lab (micro-meso) materials | Spray-drying process | - Si and Al sources - TPAOH - F127 - ethanol | **Low-high**  non-uniform  shape  7.2–26 nm | **High** | **High** | **Medium**   - 18 h preparation - Few seconds for synthesis - 8 h calcination | **Medium**   - Preparation and synthesis at 25 and 350 ºC - Calcination at 550 ºC | | **Medium**   - Expensive (templates) - High energy consumption due to length synthesis | | ([Pega et al., 2009](#_ENREF_4)) | | |
| Mesoporous aluminosilicate  xerogels | Non-hydrolytic sol-gel (NHSG) | - Al(NMe_2_)_3_ - Si(OAc)_4_ - toluene - P123 - P127 | **Low-high**  non-uniform  shape  2.4–6.1 nm | **N/A** | **High** | **High**   - 12 h preparation - 7 days synthesis - 3 h calcination | **High**   - Preparation and synthesis at 80 ºC - Calcination at 550 ºC | | **High**   - Expensive (templates) - High energy consumption due to length synthesis - Complicated preparation as Al(NMe_2_)_3_ is highly flammable. - N_2_ is necessary for preparation | | ([Skoda et al., 2016](#_ENREF_5)) | | |
| Mesostructured  aluminosilicate  nanoparticles | HS | - Si and Al sources - CTAB and PEG-4000 (as binary templates) | **High**  Nanopar-ticles  2.45 nm | **N/A** | **High**  due to the use of templates | **High**   - 24 h preparation - 48 h synthesis - 6 h calcination | **High**   - Preparation and synthesis at 25 and 110 ºC - Calcination at 550 ºC | | **High**   - Expensive (templates) - High energy consumption due to length synthesis | | ([Zhai et al., 2007](#_ENREF_6)) | | |
| MZM (HT) | Top-down  (HT) | - Zeolite Y - EDTA - NaOH | **Medium**  non-uniform  pore sizes  2–20nm | **High** | **N/A** | **Medium**   - 6 h hydrothermal treatment - 30 min work-up | **Medium**   - Treatments at 100 (6 h) and 65 ºC - Calcination at 450 ºC | | **Relatively low**   - No templates - Relatively high energy consumption due to length synthesis | | This  work | | |
| MZM (MW) | Top-down  (MW) | - Zeolite Y - EDTA - NaOH | **Medium**  non-uniform  sizes 2–10 nm | **High** | **N/A** | **Low**   - 1 min MW treatment - 30 min work-up | **Low**   - Treatments at 100 (1 min) and 65 ºC - Calcination at 450 ºC | | **Relatively low**   - No templates - Low energy consumption due to quick treatment | | This  work | | |

Notes: N/A: not applicable due to insufficient information/examples/studies reported to make an assessment; HS: hydrothermal synthesis; HT: hydrothermal treatment under reflux; MW: microwave treatment; the level of mesoporosity of materials under comparison is assessed based on the external surface area, accordingly, ‘low’ as < 200 m^2^ g^−1^, ‘medium’ as between 200–600 m^2^ g^−1^, ‘high’ as > 600 m^2^ g^−1^; the toxicity is assessed based on the relevant safety data sheet from Sigma-Aldrich.

**Preliminary economic costing for the preparation of MZMs compared with MCM-41, at the laboratory scale.**

**(based on 1 gram of the materials using the currency of the British pound sterling).**

## *Cost of chemicals and materials (based on their retail prices)*

- Cost of the starting material (zeolite Y) from Zeolyst International:

1000 gm = £200 → 1 gm = 20 pence

- Cost of Ethylenediaminetetraacetic acid (EDTA) from Sigma-Aldrich:

1000 gm = £87.3 → 1 gm = 8.7 pence

- Cost of sodium hydroxide (NaOH) from Sigma-Aldrich:

2500 gm = £175 → 1 gm = 7 pence

- Cost of silica – mesostructured MCM-41 type (used for comparison) from Sigma-Aldrich:

25 gm = £463 → 1 gm = £18.52

NB: The cost calculation does not include the price of the deionised water used in the preparation.

# *Preparation cost of the MZMs*

## *Cost of the used reagents for the preparation*

- 3.334 g starting material (Y zeolite) was used in the experiment.

So, the relevant cost of 3.334 g Y zeolite = 66.68 pence…….. (A)

- 2.922 g EDTA was used for dealumination.

So, the relevant cost of 2.922 g EDTA = 25.42 pence……….. (B)

- 0.48 g NaOH was used for alkaline wash.

So, the relevant cost of 0.48 g NaOH = 3.36 pence………….. (C)

## *Cost of the energy consumption for the MZMs preparation (based on the power recorded by the plug-in power meter during the preparation)*

The following readings of the cumulative energy consumption of different treatments were recorded by a plug-in power meter,

## For the hydrothermal treatment (HT): 1.05 kWh

## For the microwave-assisted treatment (MW): 0.05 kWh

## For the alkaline treatment: 0.15 kWh

The cost of the energy consumption was calculated according to Eq. S1.

| $\text{Cost of energy consumption =} \text{used power (kWh) }\times\text{ rate (}\frac{\text{Pence (p) }}{\text{Kilowatt hour (kWh)}}\text{)}$ | S1 |
| --- | --- |

Cost of energy consumption for the MW method = 0.05 kWh × 13.85 P/kWh = 0.69 pence …………. (D)

Cost of energy consumption for the alkaline treatment = 0.15 kWh × 13.85 P/kWh = 2.07 pence …………. (E)

Therefore, the total preparation cost of the MZM-MW-1m-100 was estimated by summing the costs of reagents required and the energy consumption of the MW method.

Based on the procedure used in this work, the preparation cost of MZM-MW-1m-100 = A + B + C + D + E = 66.68 + 25.42 + 3.36 + 0.69 + 2.07 = 98.22 Pence

**The preparation cost of MZM-MW-1m-100 = 98.22 Pence = £0.98**

Cost of energy consumption for the HT method = 1.05 kWh × 13.85 P/kWh = 14.54 pence …………. (F)

Therefore, the total preparation cost of the MZM-HT-6h-100 was estimated by summing the costs of reagents required and the energy consumption of the HT method.

Based on the procedure used in this work, the preparation cost of MZM-HT-6h-100 = A + B + C + E + F = 66.68 + 25.42 + 3.36 +2.07+ 14.54 = 112.07 Pence

**The preparation cost of MZM-HT-6h-100 = 112.07 Pence = £1.12**

The cost of the prepared mesoporous materials was much lower than the retail price of MCM-41 (*i.e.* £0.9, £1.1 and £18.52 for MZM-MW-1m-100, MZM-HT-6h-100 and MCM-41, respectively).

% Reduction in the preparation time = ((6 h for HT – 0.016 h for MW) / 6 h for HT) × 100

**% Reduction in experiment time = 99.7%**

Therefore, the preparation time of MZMs was reduced by 99.7% when the MW method was used instead of the conventional HT method.

% Reduction in the cost of the used energy = ((14.54 p for HT – 0.69 p for MW) / 14.54 for HT)) ×100

**% Reduction in the cost of the used energy = 95.2 %**

The cost of energy consumption was reduced by 95.2 % when the MW method was used instead of the conventional HT method for the preparation of MZMs.

**References**

Beck, J.S., Vartuli, J.C., Roth, W.J., Leonowicz, M.E., Kresge, C.T., Schmitt, K.D., Chu, C.T.W., Olson, D.H., Sheppard, E.W., Mccullen, S.B., Higgins, J.B., and Schlenker, J.L. (1992). A new family of mesoporous molecular sieves prepared with liquid crystal templates. *Journal of the American Chemical Society* 114**,** 10834-10843.

Han, Y., Xiao, F.S., Wu, S., Sun, Y.Y., Meng, X.J., Li, D.S., Lin, S., Deng, F., and Ai, X.J. (2001). A novel method for incorporation of heteroatoms into the framework of ordered mesoporous silica materials synthesized in strong acidic media. *Journal of Physical Chemistry B* 105**,** 7963-7966.

Lee, H.I., Park, H.J., Park, Y.-K., Hur, J.Y., Jeon, J.-K., and Kim, J.M. (2008). Synthesis of highly stable mesoporous aluminosilicates from commercially available zeolites and their application to the pyrolysis of woody biomass. *Catalysis Today* 132**,** 68-74.

Pega, S., Boissiere, C., Grosso, D., Azais, T., Chaumonnot, A., and Sanchez, C. (2009). Direct aerosol synthesis of large-pore amorphous mesostructured aluminosilicates with superior acid-catalytic properties. *Angew Chem* 121**,** 2822-2825.

Skoda, D., Styskalik, A., Moravec, Z., Bezdicka, P., Babiak, M., Klementova, M., Barnes, C.E., and Pinkas, J. (2016). Novel non-hydrolytic templated sol–gel synthesis of mesoporous aluminosilicates and their use as aminolysis catalysts. *RSC Advances* 6**,** 24273-24284.

Zhai, S.R., He, C.S., Wu, D., and Sun, Y.H. (2007). Hydrothermal synthesis of mesostructured aluminosilicate nanoparticles assisted by binary surfactants and finely controlled assembly process. *Journal of Non-Crystalline Solids* 353**,** 1606-1611.

Zhang, Z., Han, Y., Zhu, L., Wang, R., Yu, Y., Qiu, S., Zhao, D., and Xiao, F.S. (2001). Strongly Acidic and High-Temperature Hydrothermally Stable Mesoporous Aluminosilicates with Ordered Hexagonal Structure *Angew Chem Int Ed Engl* 40**,** 1258-1262.

Zhao, D., Nie, C., Zhou, Y., Xia, S., Huang, L., and Li, Q. (2001). Comparison of disordered mesoporous aluminosilicates with highly ordered Al-MCM-41 on stability, acidity and catalytic activity. *Catalysis Today* 68**,** 11-20.

Zhao, D., Sun, J., Li, Q., and Stucky, G.D. (2000). Morphological Control of Highly Ordered Mesoporous Silica SBA-15. *Chemistry of Materials* 12**,** 275-279.
